# *QuickStats: *Percentage* of Adults Aged 20–64 Years with a Blood Cholesterol Check by a Health Professional^†^ During the Past 12 Months, by Poverty Status^§^ — National Health Interview Survey, 2012 and 2017^¶^

**DOI:** 10.15585/mmwr.mm6748a6

**Published:** 2018-12-07

**Figure:**
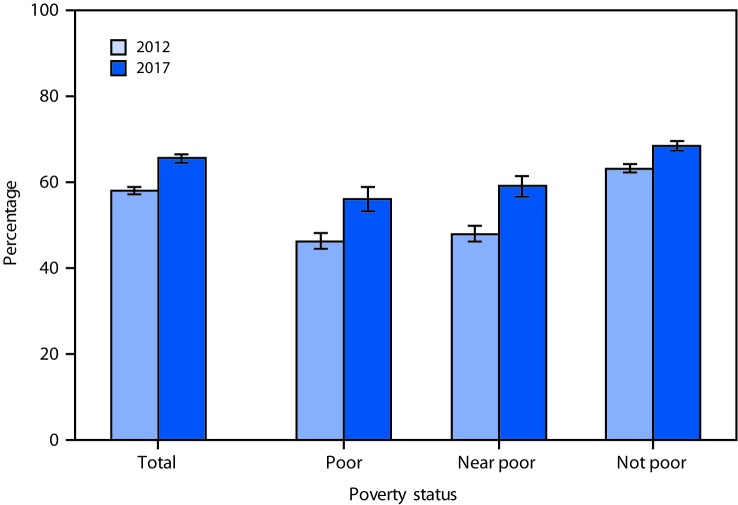
The percentage of adults aged 20–64 years who had a blood cholesterol check by a health professional in the past 12 months increased from 58.0% in 2012 to 65.5% in 2017. From 2012 to 2017, there was an increase in the percentage of adults with a blood cholesterol check among poor (46.3% to 56.0%), near poor (47.9% to 59.0%), and not poor (63.2% to 68.5%) adults. In both years, not poor adults were more likely than poor and near poor adults to have had a blood cholesterol check.

**Source:** National Health Interview Survey, 2012 and 2017 data. https://www.cdc.gov/nchs/nhis.htm.

